# Altered Expression Profile of Renal **α**_1D_-Adrenergic Receptor in Diabetes and Its Modulation by PPAR Agonists

**DOI:** 10.1155/2014/725634

**Published:** 2014-03-17

**Authors:** Xueying Zhao, Yuanyuan Zhang, Michelle Leander, Lingyun Li, Guoshen Wang, Nerimiah Emmett

**Affiliations:** Department of Physiology, Morehouse School of Medicine, Atlanta, GA 30310, USA

## Abstract

Alpha_1D_-adrenergic receptor (*α*_1D_-AR) plays important roles in regulating physiological and pathological responses mediated by catecholamines, particularly in the cardiovascular and urinary systems. The present study was designed to investigate the expression profile of *α*_1D_-AR in the diabetic kidneys and its modulation by activation of peroxisome proliferator-activated receptors (PPARs). 12-week-old Zucker lean (ZL) and Zucker diabetic fatty (ZD) rats were treated with fenofibrate or rosiglitazone for 8–10 weeks. Gene microarray, real-time PCR, and confocal immunofluorescence microscopy were performed to assess mRNA and protein expression of *α*_1D_-AR in rat kidney tissue. Using microarray, we found that *α*_1D_-AR gene was dramatically upregulated in 22-week-old ZD rats compared to ZL controls. Quantitative PCR analysis verified a 16-fold increase in *α*_1D_-AR mRNA in renal cortex from ZD animals compared to normal controls. Chronic treatment with fenofibrate or rosiglitazone reduced renal cortical *α*_1D_-AR gene. Immunofluorescence staining confirmed that *α*_1D_-AR protein was induced in the glomeruli and tubules of diabetic rats. Moreover, dual immunostaining for *α*_1D_-AR and kidney injury molecule-1 indicated that *α*_1D_-AR was expressed in dedifferentiated proximal tubules of diabetic Zucker rats. Taken together, our results show that *α*_1D_-AR expression is upregulated in the diabetic kidneys. PPAR activation suppressed renal expression of *α*_1D_-AR in diabetic nephropathy.

## 1. Introduction

Alpha_1_-adrenergic receptors (*α*_1_-ARs) are a heterogeneous family of G-protein-coupled receptors that present in most human and animal tissues, and there are considerable variations in the expression levels of *α*_1_-AR subtypes in various tissues of different species [1–4]. *α*_1_-ARs play important roles in regulating physiological and pathological responses mediated by catecholamines, particularly in the cardiovascular and urinary systems. In addition to mediating catecholamine-induced vasoconstriction, the *α*_1_-ARs in vascular wall have been shown to promote proliferation and hypertrophy of arterial smooth muscle cells and adventitial fibroblasts [5, 6]. In the urinary system, the demonstration of *α*_1_-AR expression in human prostate, bladder muscle, and smooth muscles has led to the treatment of bladder outlet obstruction and ureteral stones, via blocking these receptors [7, 8].

Of the three *α*_1_-AR subtypes (*α*_1A_, *α*_1B_, and *α*_1D_), the *α*_1D_-AR has been the least studied due to difficulties in obtaining significant expression levels and poor coupling to membrane signals due to its intracellular localization [9–11]. However, recent experiments performed in *α*_1D_-AR knockout models suggest that this *α*_1_-AR subtype plays an important role in the overall regulation of blood pressure [12]. Additionally, Armenia et al. reported that *α*_1A_- and *α*_1D_-ARs are the major functional subtypes of renal *α*_1_-ARs in both normal and streptozotocin-induced diabetic Sprague-Dawley rats [13].

Using in situ hybridization, Kurooka et al. identified the gene expression of all three *α*_1_-AR subtypes in human kidney cortex [14]. They found that intense *α*_1_-AR mRNA staining was apparent especially in the smooth muscle of arterial walls, whereas weak staining of each of the *α*_1_-AR mRNAs was observed in the glomeruli and renal tubules [14]. More recently, the presence and distribution of subtypes in human renal pelvis and calyces were evaluated, and *α*_1D_-AR was most dense in both followed by *α*_1A_ and *α*_1B_ [8]. However, the expression and distribution patterns of *α*_1D_-AR in normal and diabetic rat kidneys remain unknown.

In the present study, we examined mRNA levels of three *α*_1_-AR subtypes in the kidney cortex of normal Zucker lean (ZL) and Zucker diabetic (ZD) rats by microarray and real-time PCR analyses. Confocal immunofluorescence microscopy was performed to evaluate the distribution of *α*_1D_-AR in the kidney of Zucker rats. Furthermore, we investigated whether activation of peroxisome proliferator-activated receptor-*α* (PPAR*α*) or PPAR*γ*, known renoprotective intervention in animal models of type 2 diabetes, would affect renal expression of *α*_1_-AR subtypes.

## 2. Materials and Methods

### 2.1. Experimental Animals

Six-week-old male Zucker lean (ZL) and Zucker diabetic fatty (ZD) rats were purchased from Charles River Laboratories (Wilmington, MA, USA). Rats were housed in a temperature-controlled room with a 12 : 12-hour light-dark cycle and free access to Purina 5008 rat chow and water. Blood glucose was monitored using the Accu-chek glucometer by tail-vein blood sampling. In a previous study in which we characterized the time course of blood glucose in this model [15], we showed that blood glucose of the ZD rats began to increase at week 8, reached a peak at week 12, and remained at this higher level thereafter. The rats were housed in the animal care facility at the Morehouse School of Medicine that is AAALAC accredited. All animal protocols were approved by the Institutional Animal Care and Use Committee and were in accordance with the requirements stated in the National Institutes of Health Guide for the Care and Use Laboratory Animals.

For fenofibrate treatment, 12-week-old Zucker rats were divided into 3 experimental groups: vehicle- (0.5% carboxymethylcellulose ig) treated ZL, vehicle-treated ZD, or fenofibrate- (150 mg/kg/day ig) treated ZD (F-ZD) rats for 10 weeks. For rosiglitazone treatment, 12-week-old ZD animals were treated with rosiglitazone (10 mg/kg/day in drinking water) or vehicles for 8 weeks.

### 2.2. Isolation of Glomeruli

Glomeruli were isolated from the kidney cortex of ZL and ZD rats at the age of 7, 12, and 20–22 weeks, respectively, by a modified procedure as described previously [16]. Briefly, the rats were anesthetized and the kidneys were rapidly removed and placed in Hanks' balanced salt solution (HBSS) at pH 7.4. The renal cortex was dissected and cut into small pieces with a surgical blade. Glomeruli were isolated by passing the tissues successively through calibrated sieves (pore size: 200, 125, and 65 *μ*m) and rinsed with HBSS. Isolated glomeruli, collected on the 65 *μ*m sieve, were resuspended in HBSS. They were devoid of Bowman's capsule. Tubular fragments were less than 3% of the total number of isolated glomeruli [16].

### 2.3. RNA Extraction

Total RNA was prepared from isolated glomeruli or kidney cortex by using ultrapure TRIzol reagent according to the manufacturer's instructions (GIBCO-BRL, Grand Island, NY). The quality of the RNA samples was assessed using the Agilent 2100 Bioanalyzer (G2938A). RNA concentrations were determined spectrophotometrically (absorbance at 260 and 280 nm).

### 2.4. Microarray Analysis

Seven hundred and fifty nanograms of total RNA per sample were used for cRNA synthesis and amplification. Agilent Spike-In control RNA was included as an internal control. Cyanine-3-(Cy3-) labeled cRNA was purified and hybridized to Agilent Whole Rat Genome 44k Oligo Microarray chips (p/n G2519F-14870, Agilent Technologies) according to the manufacturer's instructions. The processed microarrays were scanned with the Agilent G2565BA DNA Microarray Scanner (p/n G2505-A). The scanned images were analyzed with Agilent Feature software (version 9.5.1.1) using default parameters. The resulting text files were loaded into the Agilent GeneSpring GX software (version 7.3) for further analysis. Gene expression values of all datasets were normalized using median normalization. Significantly differentially expressed *α*-adrenergic receptor genes among ZL, ZD, and F-ZD groups were identified by a threshold of ≥2 fold change and *P* ≤ 0.05.

### 2.5. Real-Time PCR for *α*_1A_-, *α*_1B_-, and *α*_1D_-AR mRNA Expression

Reverse transcription was performed on equal amounts of total RNA by using random hexanucleotide primers to produce a cDNA library for each sample. Real-time PCR reactions were run on an iCycler iQ Real-Time PCR Detection System by using Taqman Universal PCR Master Mix (Applied Biosystems, P/N 4304437). *α*_1A_-, *α*_1B_-, and *α*_1D_-AR and *β*-actin gene-specific Taqman probe and primer sets were obtained from Applied Biosystems as Assays-on-Demand (AOD) gene expression products. The AOD identification numbers were Rn00567876 for *α*_1A_-AR, Rn01471343 for *α*_1B_-AR, Rn00577931 for *α*_1D_-AR, and 4331182 for rat *β*-actin endogenous control. Each sample was run in triplicate, and the comparative threshold cycle (C_t_) method was used to quantify fold increase (2^−ΔΔC_t_^) compared with controls.

### 2.6. Immunostaining

For immunofluorescent staining, 5 *μ*m thick cryostat sections of OCT-embedded kidney samples were used. To study the localization of *α*_1D_-AR in the rat kidney, the sections were incubated with a mixture of two antibodies overnight: rabbit anti-*α*_1D_-AR antibody (1 : 100, Sigma-Aldrich, St. Louis, MO), mouse anti-*α*-smooth muscle actin (*α*-SMA, 1 : 100, Santa Cruz Biotechnology, Dallas, TX), or goat anti-kidney injury molecule-1 (Kim-1, 1 : 100, R&D Systems, Minneapolis, MN). As a negative control, the sections were exposed to nonimmune IgG (in replacement of primary antibodies) with the same secondary antibodies, and no specific staining occurred. The sections were observed and imaged by Leica confocal microscope.

### 2.7. Statistical Analysis

Data are expressed as mean ± SEM. Student's *t*-test was used for comparison between the two groups. Comparisons among multiple groups were performed by one-way ANOVA and Newman-Keuls post hoc test. *P* < 0.05 was considered statistically significant.

## 3. Results

### 3.1. Differential Gene Expression of Alpha-Adrenergic Receptor Subtypes in the Diabetic Kidney

We performed microarray analysis to assess gene expression levels of *α*-adrenergic receptor subtypes in the kidney cortex of 22-week-old normal ZL (blood glucose: 108 ± 8 mg/dL) and diabetic ZD (blood glucose: 425 ± 35 mg/dL) rats. The expression profile of each experimental group was determined in three animals per group.  Table 1 shows gene expression levels of *α*_1_- and *α*_2_-adrenergic receptor subtypes detected in rat kidney tissue. The rank order of expression levels of the three *α*_1_-AR mRNAs in normal rat kidney cortex was *α*_1B_ > *α*_1D_ > *α*_1A_. Among these genes, *α*_1D_-AR was significantly upregulated in the kidney cortex of diabetic animals. Compared to normal ZL controls, renal cortical *α*_1D_-AR gene was increased by 553% in 22-week-old ZD rats (Table 1).

**Figure 1:**
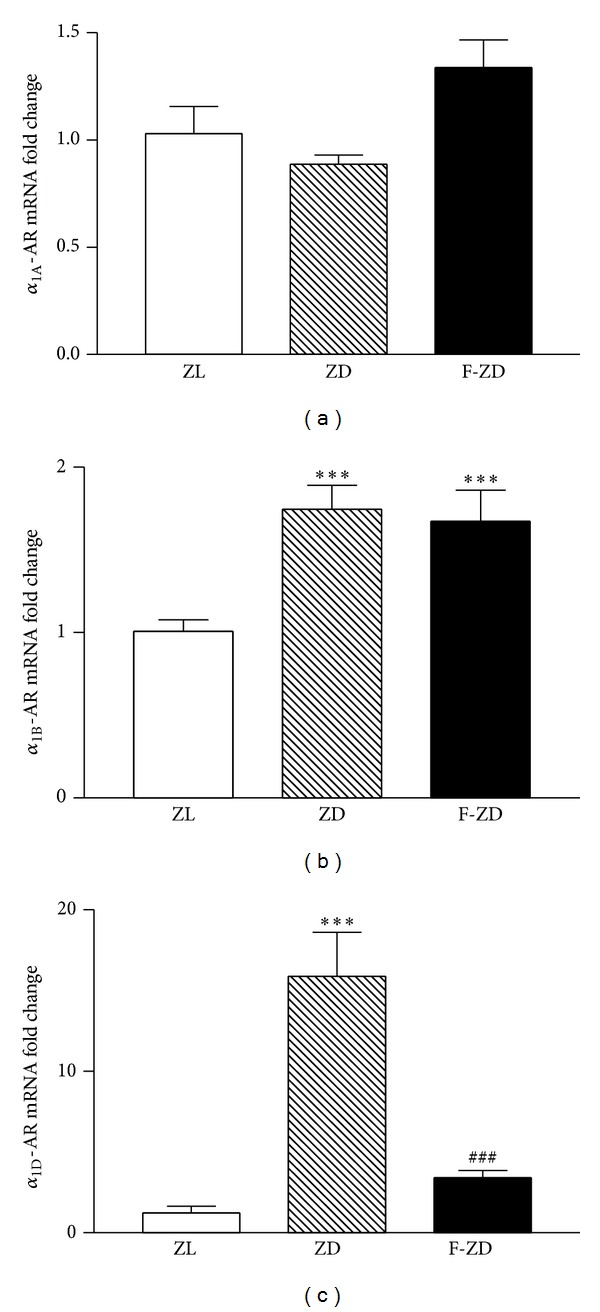
mRNA expression of renal cortical *α*_1A_-, *α*_1B_-, and *α*_1D_-AR subtypes was measured by quantitative real-time PCR in 22-week-old Zucker lean (ZL), vehicle-treated Zucker diabetic (ZD), and fenofibrate-treated Zucker diabetic (F-ZD) rats. Fold changes of *α*_1A_- (a), *α*_1B_- (b), and *α*_1D_- (c) AR genes were calculated using *β*-actin as an internal control. Values are mean ± SEM. *n* = 5-6 animals/group. ***P < 0.001 versus ZL; ^###^ *P* < 0.001 versus ZD group.

To verify the relative transcript levels of *α*_1A_-, *α*_1B_-, and *α*_1D_-AR subtypes derived from the microarray experiment, we performed quantitative real-time PCR (qPCR) assay. The relative expression levels of *α*_1A_-, *α*_1B_-, and *α*_1D_-AR mRNAs normalized against *β*-actin in rat kidney cortex are shown in Figure 1. In consistency with the microarray results, qPCR analysis showed that renal cortical *α*_1D_ mRNA was increased by 16-fold in 22-week-old ZD rats compared to ZL controls.

### 3.2. Activation of PPAR*α* or PPAR*γ* Inhibited the Upregulation of *α*_1D_-AR mRNA Expression in the Diabetic Kidneys

We have previously shown that PPAR activation protects against kidney injury in Zucker diabetic fatty rats [15, 17]. Here, we further examined the effect of PPAR activation on the expression level of *α*_1_-AR mRNAs in the diabetic kidneys. Gene microarray analysis revealed that PPAR*α* activation inhibits the upregulation of *α*_1D_ gene in the diabetic kidneys. Chronic administration of fenofibrate, a PPAR*α* agonist, resulted in a decrease in *α*_1D_-AR mRNA by 64% compared to vehicle-treated ZD animals (Table 1). qPCR analysis confirmed a reduction of *α*_1D_-AR mRNA in the kidney of F-ZD rats (Figure 1). In contrast, both microarray and qPCR analyses indicated that mRNA expression of *α*_1A_- and *α*_1B_-AR subtypes was not affected by fenofibrate in the diabetic kidneys.

**Figure 2:**
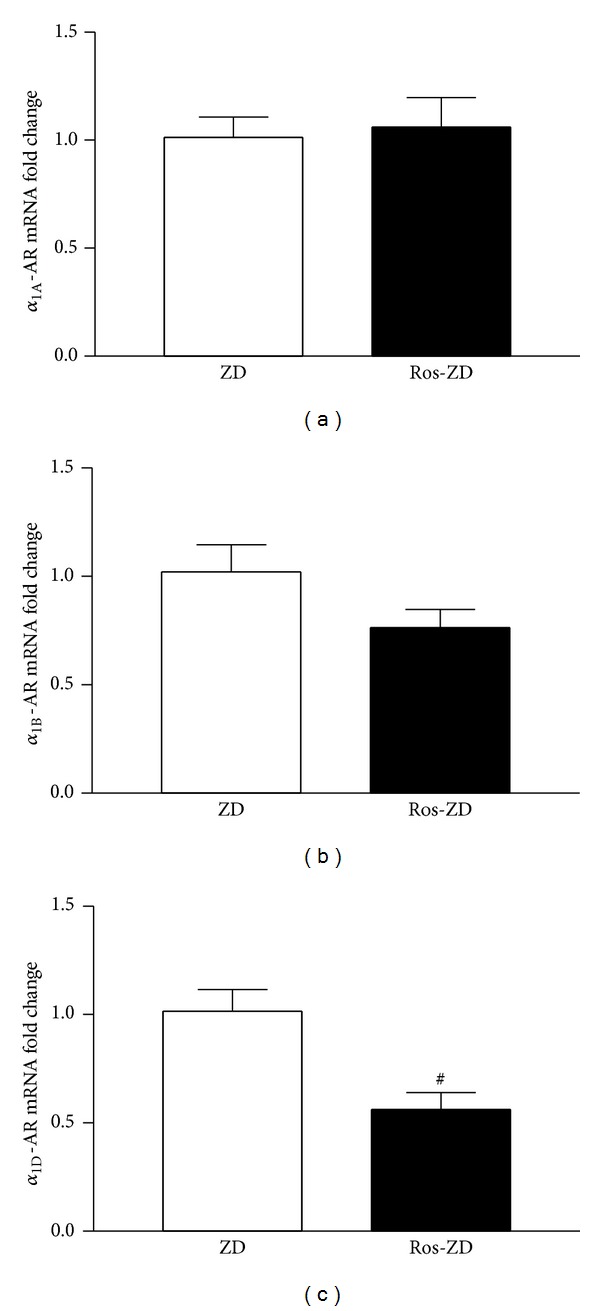
Effect of PPAR*γ* activationon renal expression of *α*_1A_-, *α*_1B_-, and *α*_1D_-AR subtype mRNAs. Bar graphs present the real-time PCR results of renal cortical *α*_1A_- (a), *α*_1B_- (b), and *α*_1D_- (c) AR mRNAs in 20-week-old ZD rats following treatment with rosiglitazone for 8 weeks. Values are mean ± SEM. *n* = 5-6 animals/group. ^#^ *P* < 0.05 versus vehicle-treated ZD group.

Additionally, the effect of PPAR*γ* activation on gene expression of *α*_1_-AR subtypes was examined in the ZD rats after rosiglitazone treatment. Compared to vehicle-treated ZD rats, renal cortical *α*_1D_-AR mRNA was significantly lower when rosiglitazone was administered for 8 weeks (Figure 2). Similar to PPAR*α* activation, *α*_1A_- and *α*_1B_-AR mRNA expression in the diabetic rats was not affected by rosiglitazone treatment.

### 3.3. Glomerular and Cortical Expression of *α*_1D_-AR mRNA in Zucker Rats of Various Ages

**Figure 3:**
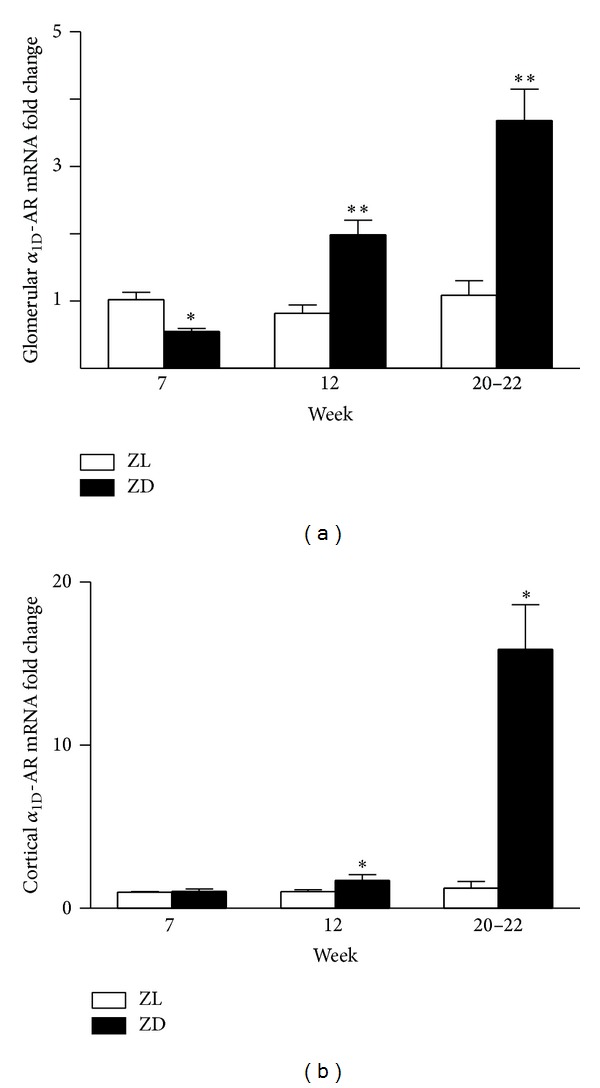
mRNA expression of *α*_1D_-AR in the glomeruli and cortex of normal ZL and diabetic ZD rats at different time points. *α*_1D_-AR gene was progressively increased in both glomeruli (a) and cortex (b) of ZD rats compared to normal ZL littermates. mRNA fold changes of *α*_1D_-AR were calculated using *β*-actin as an internal control. Values are mean ± SEM. *n* = 5-6 animals/group. *P < 0.05, **P < 0.01 versus ZL control group.

In a previous study [15], we showed that blood glucose levels were not different between normal ZL and diabetic ZD rats at the age of 7 weeks. Blood glucose of the ZD rats began to increase at week 8, reached a peak at week 12, and remained at this higher level thereafter. To analyze the temporal pattern of renal expression of *α*_1D_-AR receptors, renal cortical and glomerular *α*_1D_-AR mRNA levels were compared in the Zucker rats at the ages of 7, 12, and 20–22 weeks. As shown in Figure 3, ZD rats at week 7 had slightly lower *α*_1D_-AR mRNA level in the glomeruli compared to their ZL littermates, whereas renal cortical *α*_1D_-AR mRNAs were not different between the two groups. At the age of 12, both renal glomerular (2.4-fold) and cortical (1.7-fold) *α*_1D_-AR mRNA levels were significantly higher in the ZD animals, which were further increased by 3.4-fold and 12.9-fold, respectively, at the age of 20–22 weeks.

### 3.4. Expression and Distribution of *α*_1D_-AR Protein in the Kidney of Zucker Rats

**Figure 4:**
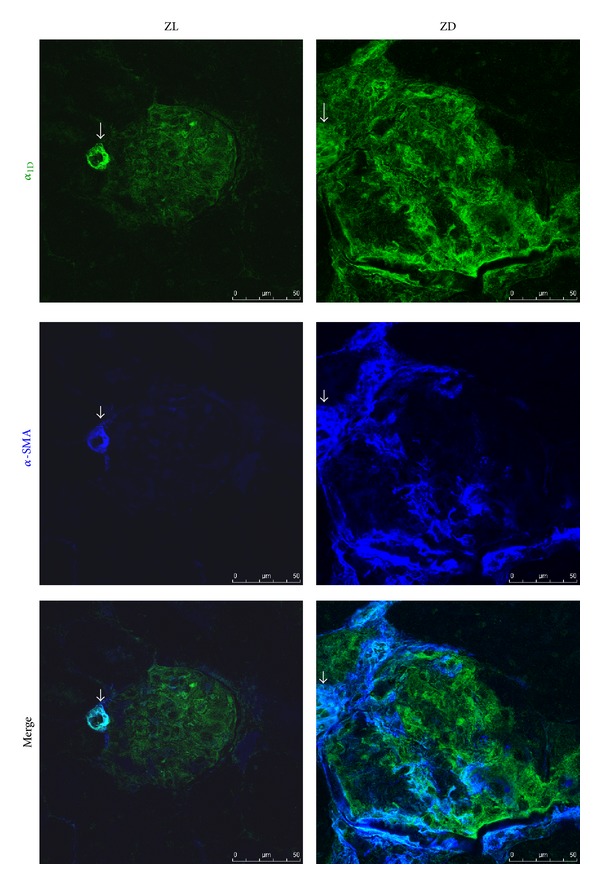
Representative confocal immunofluorescence images of *α*_1D_-AR and *α*-smooth muscle actin (*α*-SMA) in the glomeruli of ZL and ZD rats. In normal control, colocalization of *α*_1D_-AR (green) with *α*-SMA (dark blue) was apparent in the renal vasculature (white arrow), whereas weak staining of *α*_1D_-AR was detected in the glomeruli. An increase in *α*_1D_-AR staining in the diabetic glomeruli was accompanied by an increase in *α*-SMA signal.

**Figure 5:**
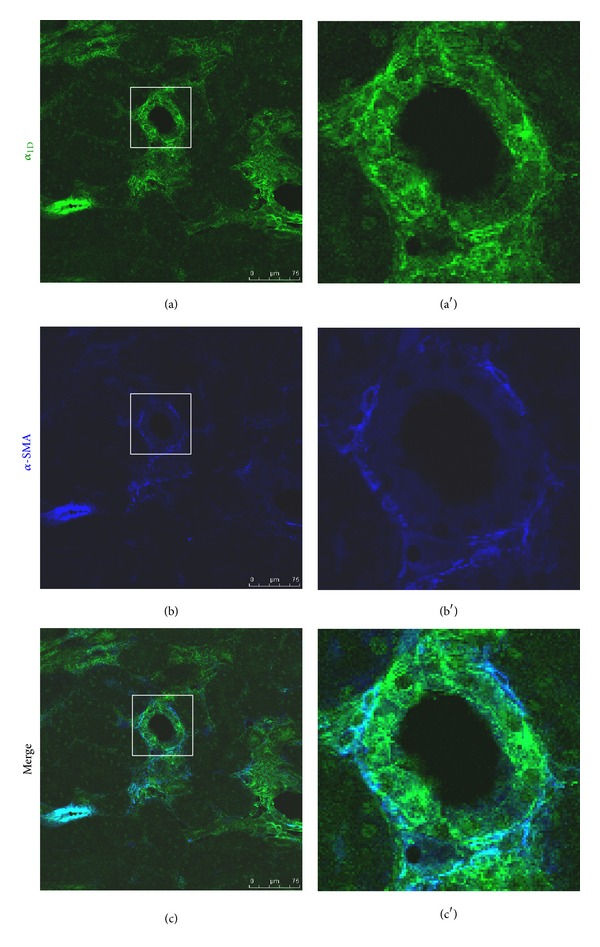
Increased tubulointerstitial *α*_1D_-AR staining in the kidney of 20-week-old Zucker diabetic rats. In the diabetic kidneys, an induction of *α*_1D_-AR protein (green) was detected in the epithelium of dilated tubules as well as the surrounding interstitial cells, which are *α*-SMA-positive (dark blue). Images in (a′), (b′), and (c′) are enlarged from the boxed areas in (a), (b), and (c).

We performed immunofluorescence staining to correlate *α*_1D_-AR gene expression results with its protein level and distribution in the kidney of Zucker animals. As expected, *α*_1D_-AR protein was clearly detected in the renal arteries and arterioles in both normal and diabetic animals (Figure 4). The intense staining was primarily in the smooth muscle of renal arterial walls as evidenced by its colocalization with *α*-SMA. In normal ZL rats, weak *α*_1D_-AR staining was also detected in the glomeruli, whereas there was no obvious staining in the renal tubules (Figure 4). In consistency with the mRNA expression results, *α*_1D_-AR protein was largely induced in the diabetic ZD kidneys. Immunofluorescence staining identified increased *α*_1D_-AR signal in the glomeruli of diabetic rats, which was partially colocalized with *α*-SMA (Figure 4). Moreover, intense tubulointerstitial staining of *α*_1D_-AR was apparent in the diabetic kidneys. As shown in Figure 5, *α*_1D_-AR was expressed in both tubular epithelial cells and activated interstitial fibroblasts, which was positive for *α*-SMA staining.

**Figure 6:**
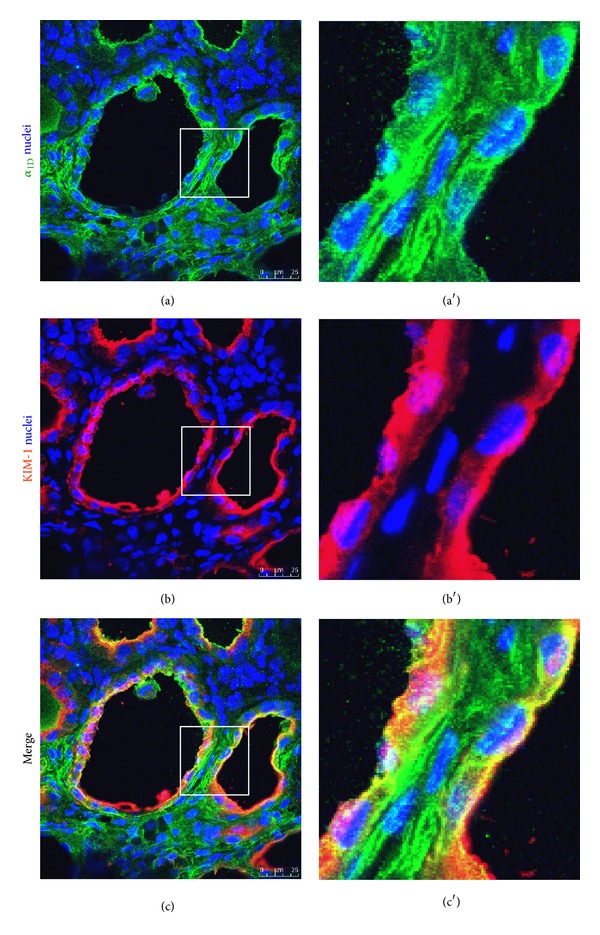
Representative confocal images of double staining for *α*_1D_-AR and Kim-1 in the tubules of 20-week-old Zucker diabetic rats. Colocalization of *α*_1D_-AR (green) and Kim-1 (red) was seen in the dilated proximal tubules of diabetic rats. Images in (a′), (b′), and (c′) are enlarged from the boxed areas in (a), (b), and (c).

To further characterize *α*_1D_-AR expression in renal tubules of diabetic animals, we performed double staining for *α*_1D_-AR and Kim-1, a sensitive tubular injury marker. Dual labeling revealed a spatial relationship between Kim-1 and *α*_1D_-AR in the diabetic kidneys. As shown in Figure 6, virtually all dilated tubules expressing *α*_1D_-AR were also Kim-1-positive, suggesting that *α*_1D_-AR was expressed in the injured dedifferentiated proximal tubules. In these tubules, *α*_1D_-AR expression was predominantly cytoplasmic, whereas Kim-1 staining was prominent at the apical membrane.

## 4. Discussion

In this study, the expression and distribution of *α*_1D_-AR mRNA and protein were determined by the gene microarray, qPCR, and confocal immunofluorescence analyses. Although mRNA expression of all three *α*_1_-AR subtypes (*α*_1B_ > *α*_1D_ > *α*_1A_) was detected in rat kidney cortex, only *α*_1D_-AR gene was massively upregulated in the diabetic animals. Moreover, diabetes-related increase in *α*_1D_-AR mRNA was inhibited when the ZD rats were treated with fenofibrate or rosiglitazone. Immunostaining for *α*_1D_-AR confirmed that intense *α*_1D_-AR staining was apparent especially in the smooth muscle of arterial walls in both normal and diabetic kidneys. Weak *α*_1D_-AR protein staining was detected in the glomeruli of normal ZL controls, but there was no obvious staining in the normal tubular epithelium. In consistency with the gene expression results, *α*_1D_-AR protein was significantly increased in the glomeruli and proximal tubules of diabetic animals.

The expression of *α*_1_-AR subtype mRNAs has previously been studied in various animal and human organs, and the predominant subtype mRNA expressed differs among species and organs. For example, Kurooka et al. reported that *α*_1A_-AR gene was detected more than *α*_1B_-AR or *α*_1D_-AR in human kidney cortex [14]. In contrast, Karabacak et al. [8] recently evaluated *α*_1_-AR subtype protein expression in human renal pelvis and calyx tissues and found that *α*_1D_-AR was most dense in both followed by *α*_1A_- and *α*_1B_-AR subtypes, respectively, where the rate of *α*_1B_-AR was significantly lower than the other two. In the rat kidney, it was reported that the *α*_1B_-AR is predominant when detected by a radioligand binding assay [18] and RNase protection assay [19]. In consistency with these findings, we confirmed the expression of all three *α*_1_-AR subtype mRNAs (*α*_1B_ > *α*_1D_ > *α*_1A_) in rat kidney cortex by microarray and qPCR analyses.

Of the three *α*_1_-AR subtypes, the *α*_1D_-AR has been the least studied due to difficulties in obtaining significant expression levels and poor coupling to membrane signals due to its intracellular localization [9–11]. However, recent experiments performed in *α*_1D_-AR knockout models suggest that this *α*_1_-AR subtype plays an important role in the overall regulation of blood pressure [12]. Moreover, Armenia et al. reported that *α*_1A_- and *α*_1D_-ARs are the major functional subtypes of renal *α*_1_-ARs in both normal and streptozotocin-induced Sprague-Dawley rats [13]. Interestingly, among the three *α*_1_-AR subtypes detected in rat kidney cortex, we found that *α*_1D_-AR mRNA was markedly increased by 16-fold in the diabetic kidneys. Additionally, diabetes-associated upregulation of *α*_1D_-AR mRNA expression was inhibited when the diabetic animals were treated with PPAR agonists, known renoprotective interventions. Therefore, previous evidences and present results strongly suggest that *α*_1D_-AR may play an important role in renal physiology and/or pathophysiology.

Diabetic nephropathy is characterized by a series of ultrastructural changes, including glomerular and tubular hypertrophy, mesangial expansion, glomerulosclerosis, and tubulointerstitial fibrosis. We have previously demonstrated a progressive loss of renal function in the diabetic animals as evidenced by an increase in urinary protein to creatinine ratio in the ZD rats between the ages of 7 and 20 weeks [15]. In the current study, we further observed a gradual increase in cortical and glomerular *α*_1D_-AR mRNA expression during disease progression in the diabetic animals. We speculate that increased *α*_1D_-AR may play a role in the development of diabetic renal hypertrophy and fibrosis. In fact, a role for *α*_1D_-AR in vascular hypertrophy and remodeling has been suggested by a recent report that a decrease in the lumen area and an increase in the wall thickness of arteries in hypoxic pulmonary hypertension were strongly inhibited in *α*_1D_-AR knockout mice [5]. In this study, we evaluated the expression and distribution of *α*_1D_-AR protein in the kidneys of ZL and ZD rats by immunofluorescence staining. As expected, intense *α*_1D_-AR staining was apparent especially in the smooth muscle of arterial walls in both normal and diabetic kidneys. Compared to low expression level of *α*_1D_-AR protein in the glomeruli of normal ZL controls, ZD rats demonstrate a significant increase in *α*_1D_-AR signal. Moreover, ZD kidneys displayed strong tubulointerstitial *α*_1D_-AR signal within fibrotic areas. *α*_1D_-AR protein was expressed in both tubular epithelial cells and interstitial cells. A colocalization of *α*_1D_-AR with *α*-SMA indicates that *α*_1D_-AR expressing interstitial cells are myofibroblasts. Future work in the field could be necessary to establish the exact role of *α*_1D_-AR in activation and proliferation of renal interstitial fibroblasts.

Another interesting novelfinding in our set of data reported here is the *α*_1D_-AR expression in tubular epithelial cells of diabetic kidneys. Although there was no obvious staining in the normal tubules, *α*_1D_-AR staining was apparent in the dilated tubules in the fibrotic areas. To further characterize *α*_1D_-AR expression in renal tubules of diabetic animals, the spatial relationship between tubular *α*_1D_-AR and Kim-1, a tubular injury marker, was studied by double staining. In tubulointerstitial fibrotic areas, virtually all dilated tubules expressing *α*_1D_-AR were also Kim-1-positive. The selective increased expression by dedifferentiated epithelial cells and activated interstitial fibroblasts supports the potential importance of *α*_1D_-AR in renal tubulointerstitial injury. Moreover, recent studies on the pulmonary circulation suggest that catecholamines may participate in the excessive muscularization and fibrosis of the arteries through ERK1/2 signaling pathway in hypoxic pulmonary hypertension [5, 20–22]. Therefore, further studies are warranted to evaluate the functional consequences of *α*_1D_-AR induction in diabetic kidney injury.

In summary, the current study highlights mRNA expression of the three *α*_1_-AR subtypes in rat kidney cortex. An increase in renal expression of *α*_1D_-AR mRNA and protein was associated with glomerular and tubulointerstitial injury in diabetic nephropathy. Chronic treatment with PPAR agonists prevents the increase in *α*_1D_-AR mRNA in the diabetic kidneys. These findings may provide new insights into the prevention and early management of diabetic kidney disease.

## Figures and Tables

**Table 1:** Alpha-adrenergic receptor gene expression in the kidney cortex of Zucker lean (ZL), untreated Zucker diabetic fatty (ZD), and fenofibrate-treated diabetic fatty (F-ZD) rats.

Accession number	Gene name	mRNA expression ZL	ZD versus ZL		F-ZD versus ZD	
			Fold change	*P* value	Fold change	*P* value
NM_017191	*α*_1A_-adrenergic receptor (*α*_1A_-AR)	10.4 ± 2.9	NA	NA	NA	NA
NM_016991	*α*_1B_-adrenergic receptor (*α*_1B_-AR)	1372.2 ± 101.8	1.49	NS	1.26	NS
NM_024483	*α*_1D_-adrenergic receptor (*α*_1D_-AR)	273.3 ± 142.9	6.53	<0.001	−2.75	<0.001
NM_012739	*α*_2A_-adrenergic receptor (*α*_2A_-AR)	53.2 ± 15.2	−1.09	NS	1.02	NS
NM_138505	*α*_2B_-adrenergic receptor (*α*_2B_-AR)	116.8 ± 22.8	−1.18	NS	1.05	NS
NM_138506	*α*_ 2C_-adrenergic receptor (*α*_2C_-AR)	33.1 ± 7.9	1.19	NS	1.07	NS

Data are shown in two ways: mRNA expression level presenting the relative abundance of different alpha-adrenergic receptor subtypes in normal rat kidney cortex and fold change showing the differential expression of these genes among ZL, ZD, and F-ZD groups. There is significant (*P* < 0.001) upregulation in renal expression of *α*_1D_-AR gene in the ZD animals, which is markedly prevented by fenofibrate treatment (*P* < 0.001). NA: not analyzed due to its low expression level in ZL, ZD, and F-ZD animals; NS: not significant.
